# Supplementary material for: ProtCID: a data resource for structural information on protein interactions
Source: Nat Commun. 2020 Feb 5;11:711. doi: 10.1038/s41467-020-14301-4 (PMC7002494; doi:10.1038/s41467-020-14301-4)
Supplement: Supplementary file 1 — Supplementary Information [file 41467_2020_14301_MOESM1_ESM.pdf]

Supplementary Information

ProtCID: A data resource for structural information on protein interactions

Xu et al.

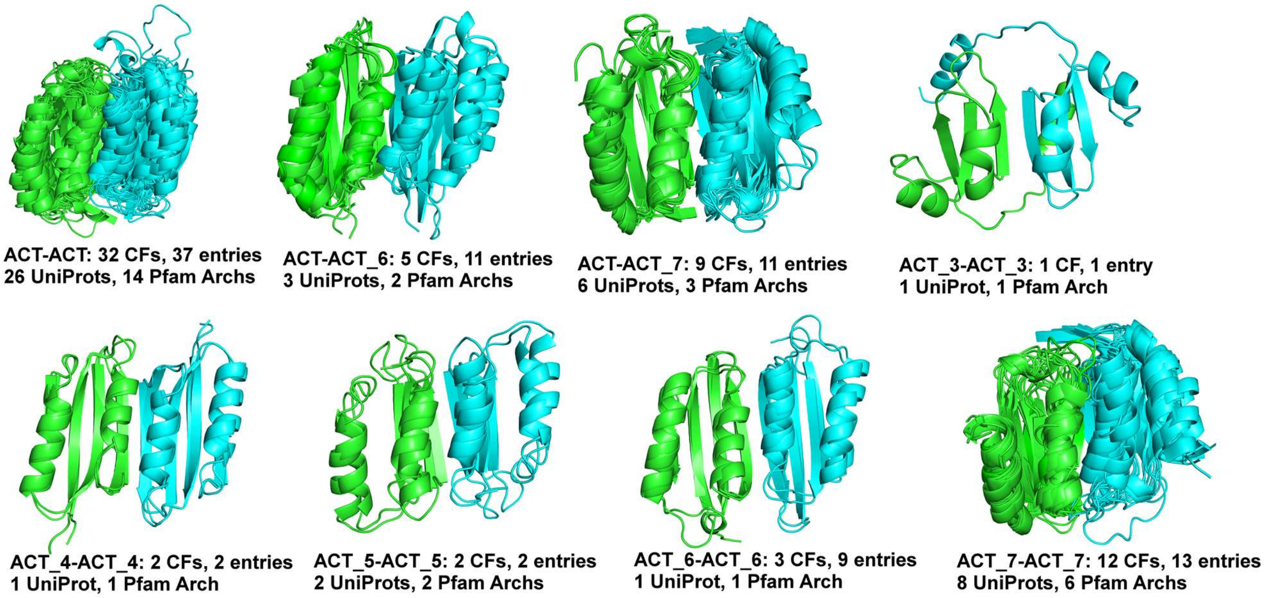

Supplementary Figure 1 | Similar homo- and heterodimers in the ACT clan, consisting of Pfams ACT, ACT_3, ACT_4, ACT_5, ACT_6, and ACT_7.

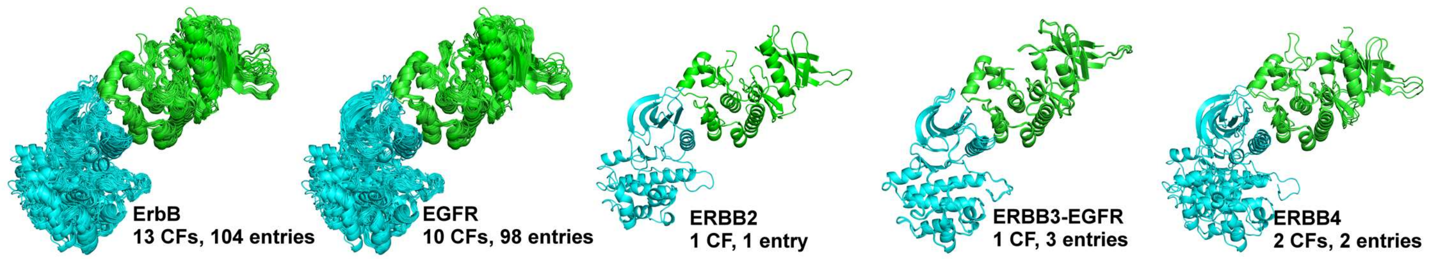

Supplementary Figure 2 | Asymmetric dimer of ErbB proteins in 13 crystal forms and 104 PDB entries. The asymmetric dimer is contained in 10/30 CFs and 98/140 entries of EGFR in the PDB, 1/2 CFs and 1/2 entries of ErbB2, 1/1 CF and 3/3 entries of heterodimers of ErbB3 (in green) and EGFR (in cyan), and 2/3 CFs and 2/4 entries of ErbB4. The numerator is the number of CFs or entries in the cluster, and the denominator is the number of total CFs or entries in the PDB. The annotation is same in all supplementary figures.

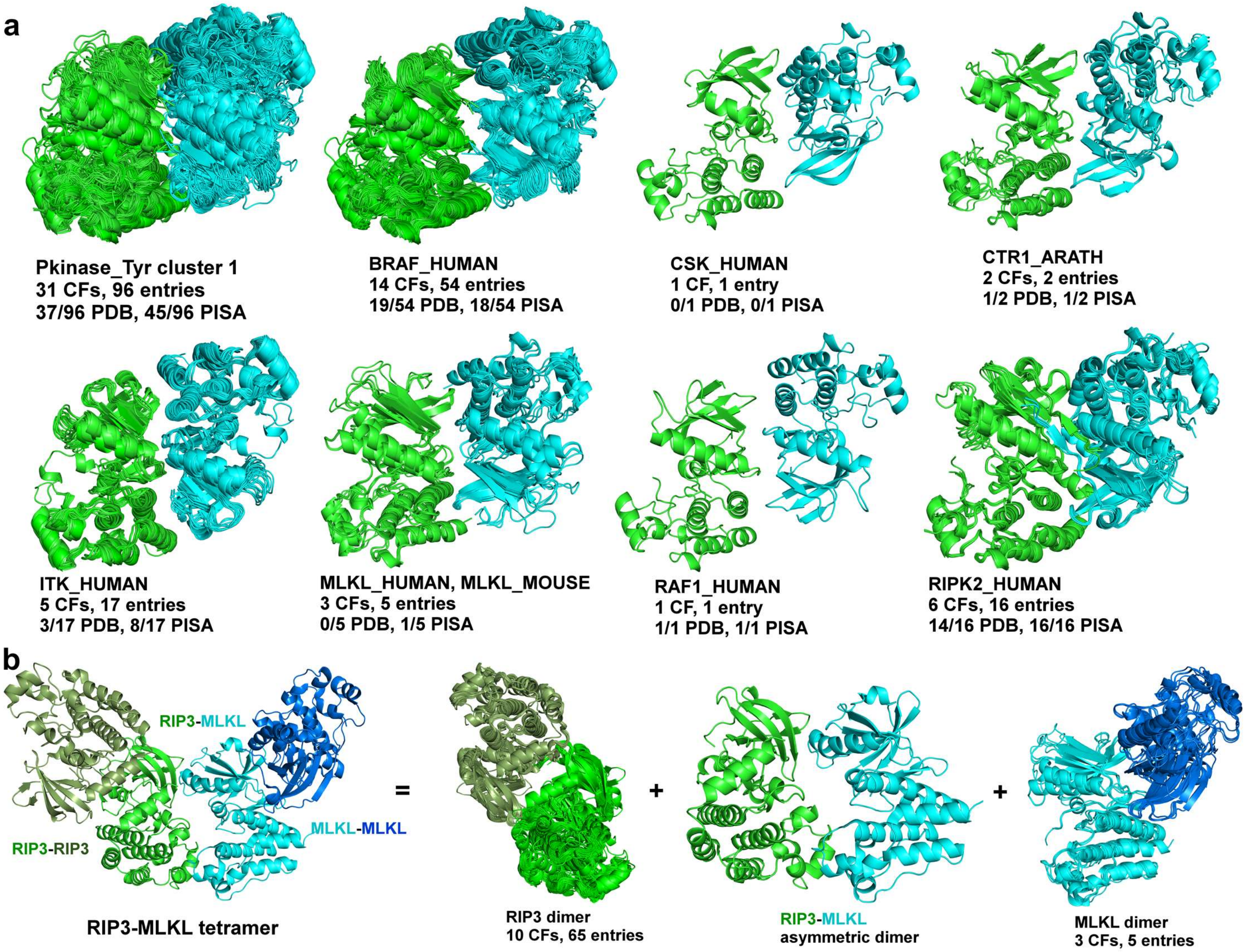

Supplementary Figure 3 | Common domain interfaces in Pkinase_Tyr (PF07714) cluster 1 in ProtCID. (**a**) The cluster contains 31 CFs and 96 entries, and exists in 37 PDB biological assemblies and 45 PISA assemblies. It contains 8 different kinases: human BRAF (14/17 CFs, 54/62 entries), CSK (1/3 CF, 1/3 entry), ITK (5/8 CFs, 17/33 entries), MLKL (2/2 CFs, 4/4 entries), RIPK2 (6/6 CFs, 16/16 entries), and RAF1 (1/1 CF, 1/1 entry); mouse MLKL (1/3 CF, 1/3 entry); and *Arapidopsis* CTR1 (2/2 CFs, 2/2 entries). (**b**) Structure of a RIPK3-MLKL kinase tetramer. The tetramer is in the crystal PDB: 4M69. In the Pfam protein classification system, RIPK3 belongs to Pfam (Pkinase) while RIPK2 belongs to Pfam (Pkinase_Tyr). RIPK3 kinase forms a symmetric dimer that is similar to that of BRAF and RIPK2 kinases. The (Pkinase) domain cluster containing RIPK3 contains 10 CFs and 65 PDB entries (domain cluster 13, including ROCK2_HUMAN (3 CFs, 4 entries); ROCK2_BOVIN (2 CFs, 2 entries); RIPK3_MOUSE (1 CF, 1 entry); and PDPK1_HUMAN (4 CFs, 58 entries). The MLKL dimer is a member of the (Pkinase_Tyr) cluster that contains BRAF and RIPK2.

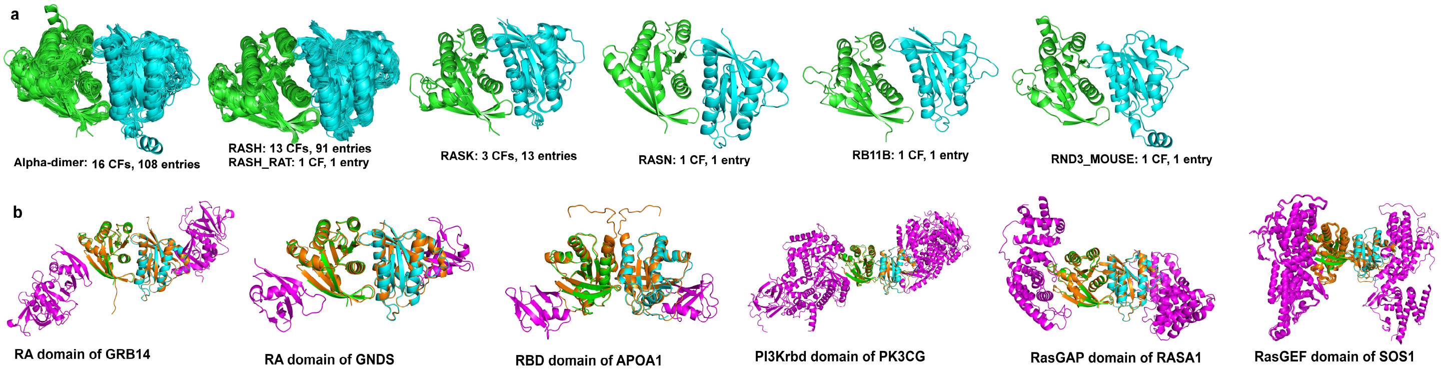

Supplementary Figure 4 | RAS dimers in ProtCID.

(**a**) Ras α4-α5 dimers of individual Ras proteins. This dimer is in 16 crystal forms and 108 entries and occurs in crystals structures of human HRAS (13/35 CFs, 89/145 entries), rat HRAS (1/1 CF, 1/1 entry), human KRAS (3/32 CFs, 13/82 entries), human NRAS (1/3 CF, 1/3 entry), human RAB11B (1/3 CF, 1/3 entry) and mouse RND3 (RhoE) (1/1 CF, 1/1 entry). (**b**) Models of other RAS heterotetramers containing the α4-α5 dimer derived from RAS/partner co-crystal structures. These crystals do not contain the α4-α5 dimer but the RAS domain in each crystal (colored in orange) could in principle bind the partner (colored in purple) and another RAS monomer simultaneously. The dimers of HRAS were built by superposition of the HRAS monomers on the dimer from PDB entry 1CLU; the dimer of KRAS was built by superposition of the KRAS monomer on the dimer from PDB entry 5VPI: 1) the RA domain of GRB14 binding to HRAS (PDB: 4K81); 2) the RA domain of RALGDS binding to HRAS (PDB: 1LFD); 3) the RBD domain of APOA1 binding to KRAS (PDB: 2MSE); 4) the PI3Krbd domain of PK3CG binding to HRAS (PDB: 1HE8); 5) the RasGAP domain of RASGAP binding to HRAS (PDB: 1WQ1); and 6) the RasGEF domains of Son-of-Sevenless bound to HRAS (41 PDB entries).

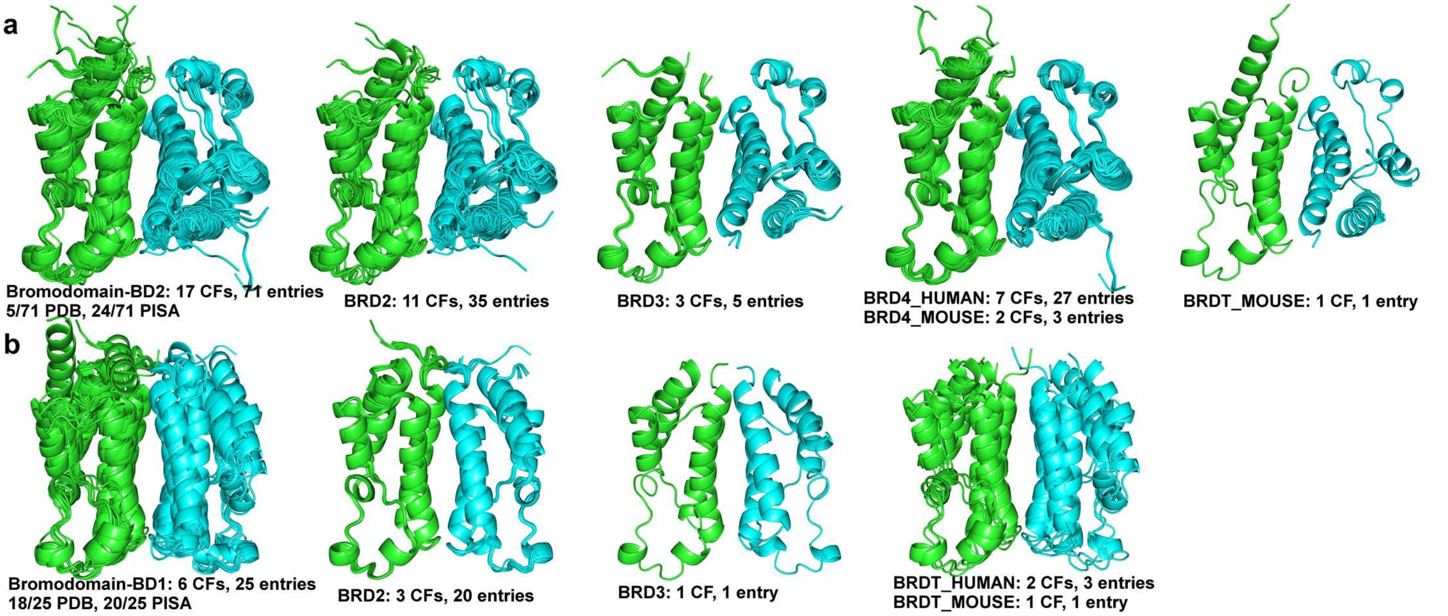

Supplementary Figure 5 | Two Bromodomain dimers of BET (Bromodomains and Extra Terminal domain) proteins. (**a**) The Bromodomain 2 (BD2) dimer of individual BET proteins. All BET BD2 crystals contain this dimer. This is a head-to-tail dimer with surface area 741 Å^2. This BD2 head-tail (H-T) dimer occurs in 17 crystal forms and 71 PDB entries, and in all four BET proteins: human BRD2 (11/11 CFs, 35/35 entries), BRD3 (3/3 CFs, 5/5 entries), and BRD4 (7/7 CFs, 27/27 entries), and mouse BRD4 (2/2 CFs, 3/3 entries) and BRDT (1/1 CF, 1/1 entry). (**b**) The Bromodomain 1 (BD1) dimer of individual BET proteins, verified by mutagenesis of BRD2 BD1 *in vivo*[1]. This is a head-to-head dimer with surface area 1015 Å^2. This dimer occurs in 6/28 CFs and 25/76 PDB entries: human BRD2 (3/18 CFs, 20/60 entries), BRD3 (1/6 CF, 1/9 entry), and BRDT (2/4 CFs, 3/5 entries) and mouse BRDT (1/2 CF, 1/2 entry).

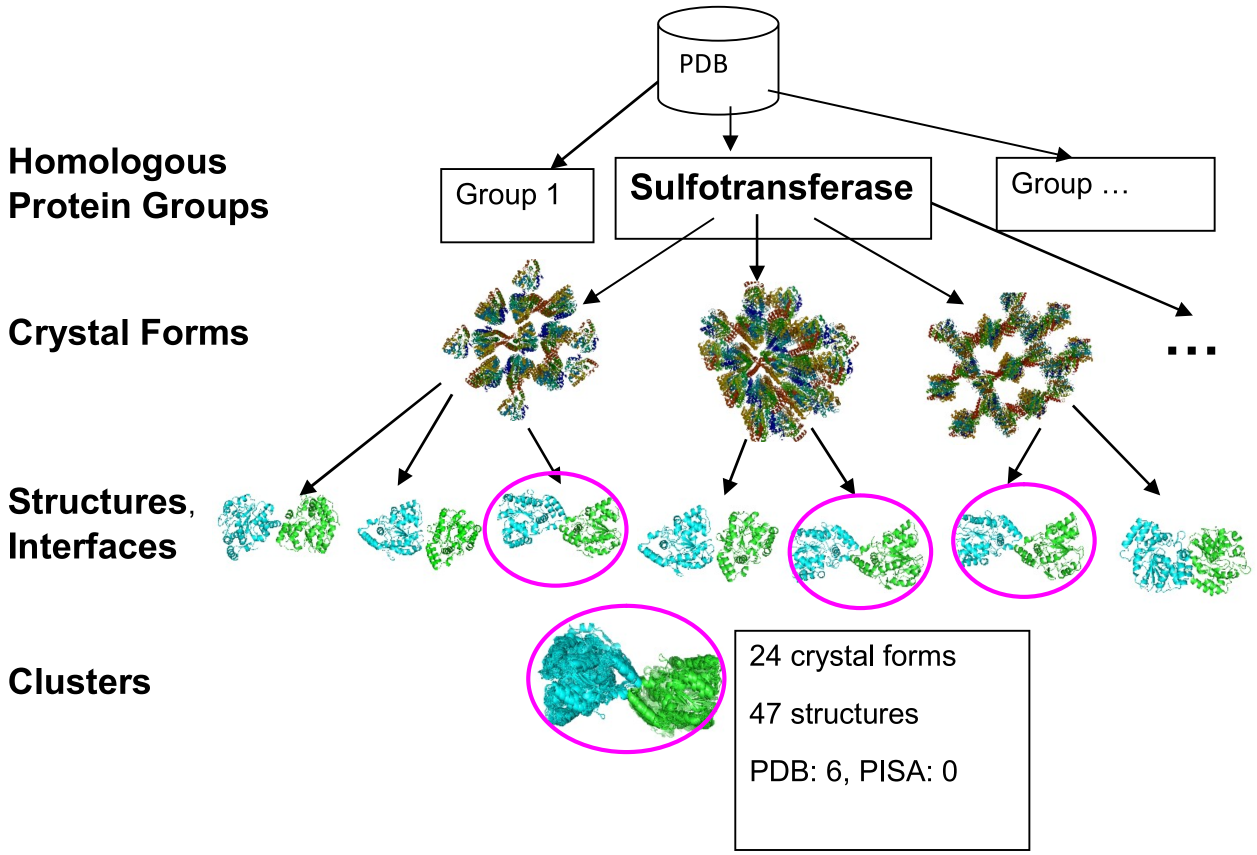

Supplementary Figure 6 | Interface clustering procedure. PDB structures are divided into homologous groups based on chain Pfam architectures or Pfam domains. For each group, the structures are divided into different crystal forms. Each NMR or cryo-EM structure is its own unique crystal form. Interfaces are calculated from crystals which are built from asymmetric units and symmetry operators stored in the PDB mmCIF files. For each crystal form, we select a representative structure with the highest resolution, and calculate the interface similarities between the representative entry and other structures in the same crystal form. We then calculate the interface similarities among the representative entries. These interfaces are clustered by average-linkage hierarchical clustering.

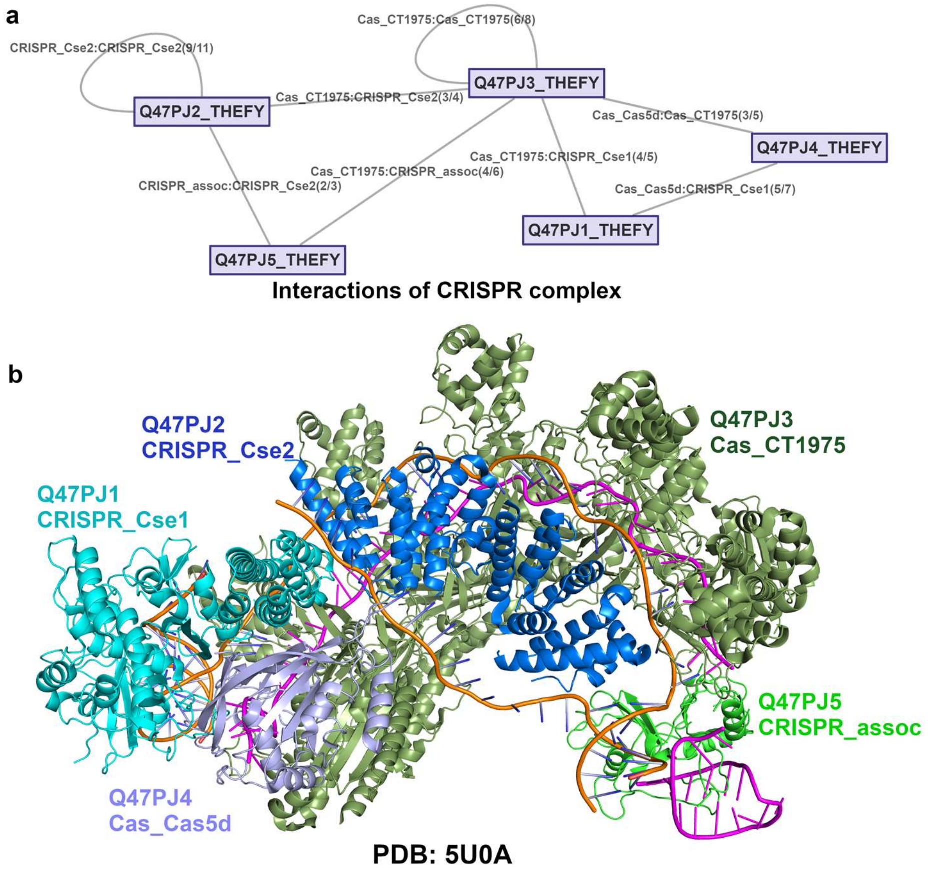

Supplementary Figure 7 | Interactions of proteins within the CRISPR cascade complex represented by UniProts and Pfams in the PDB. (**a**) The interactions returned by input of UniProts: Q47PJ1_THEFY, Q47PJ2_THEFY, Q47PJ3_THEFY, Q47PJ4_THEFY, and Q47PJ5_THEFY. An edge is labeled by Pfam:Pfam and the number of crystal forms and the number of PDB entries in the largest cluster in parentheses. (**b**) An example of CRISPR cascade complex in the PDB. Chains are marked in different colors and labeled with their UniProts and Pfams in the same colors.

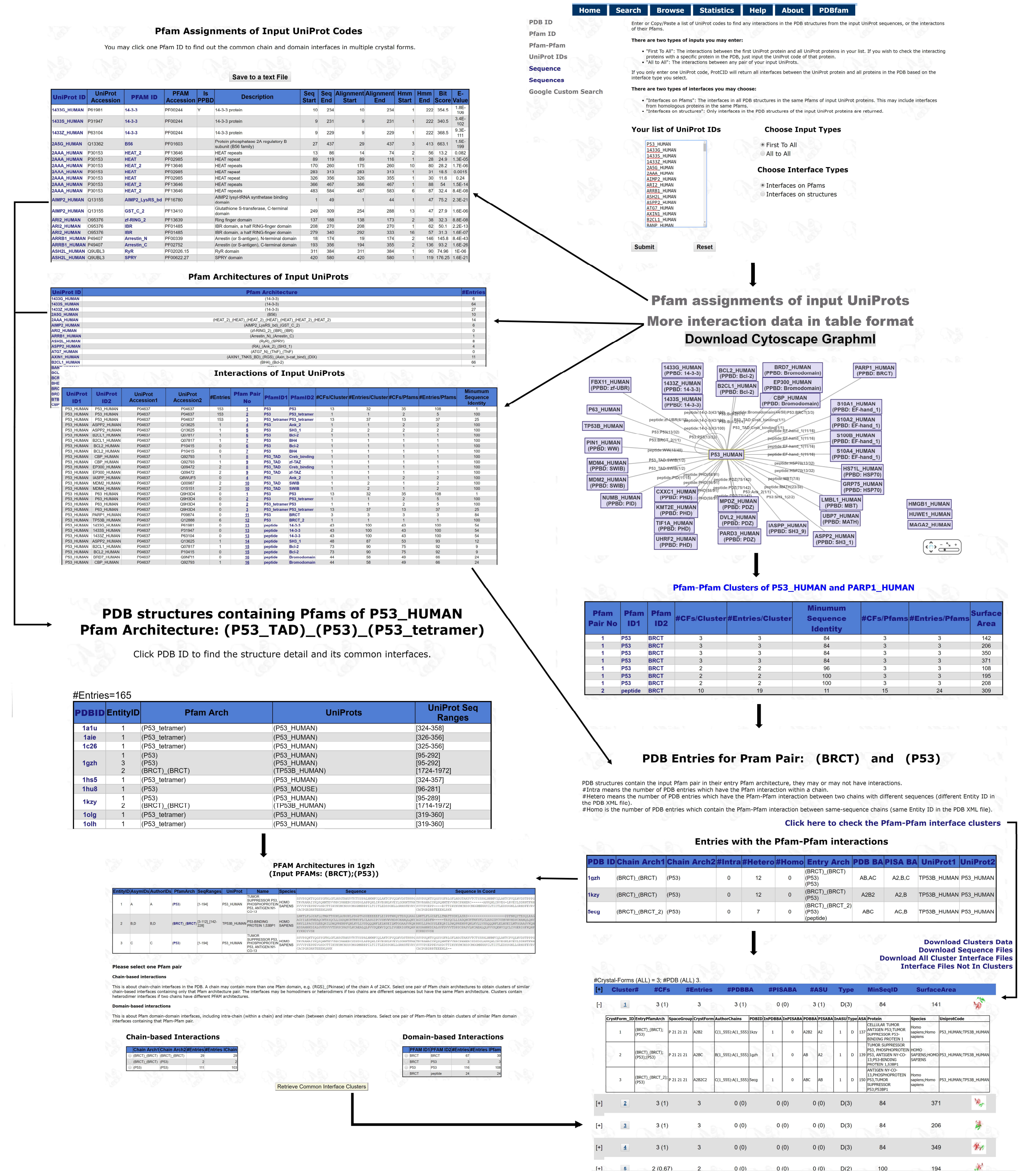

Supplementary Figure 8 | Flow chart of a web query of a hub protein and its interactors on the ProtCID web site. We used P53_HUMAN and its 33 human protein interactors that have crystal structures in the PDB. The UniProts (all appended with _HUMAN) include: P53, 1433G, 1433S, 1433Z, ASPP2, B2CL1, BCL2, BRD7, CBP, CXXC1, DVL2, EP300, FBX11, GRP75, HS71L, IASPP, KMT2E, LMBL1, MDM2, MDM4, MPDZ, NUMB, P63, PARD3, PARP1, PIN1, S100B, S10A1, S10A2, S10A4, TIF1A, TP53B, UBP7, UHRF2. “First to All” and “Interfaces on Pfams” are selected. The UniProts are assigned to Pfams, and a table is given by the link “Pfam assignments of input UniProts”. The interactions based on Pfam-Pfam are returned, and shown in a Cytoscape figure and a table via the link “More interactions in table format”. Those UniProts with no interactions with P53_HUMAN are listed separately. Clicking the P53_HUMAN node leads to a list of PDB entries containing any Pfams of P53_HUMAN, and their Pfam architectures and UniProt names. Clicking the edge between P53_HUMAN and PARP1_HUMAN leads to a table containing Pfam pairs of these two proteins, which includes the PPBD: BRCT and peptide interaction. Clicking “1” in the “Pfam Pair No” column returns two tables: (i) entries containing BRCT and P53 interaction; (ii) entries containing BRCT and P53 Pfams, but having no BRCT/P53 interaction detected in their structures. From this page, each entry can be clicked, or interface clusters can be checked by the link “Click here to check Pfam-Pfam interface clusters”.

Supplementary Table 1 | Summary of ProtCID interactions.

		Same[a]		Diff[b]		Pfam-peptide[c]	Pfam-DNA/RNA[d]	Pfam-ligand[e]
		**Chain**	**Domain**	**Chain**	**Domain**			
All	#Pfam-Archs	5319	4461	3471	6571	1083	1260	6485
	#Entries	100175	102897	11606	39204	8384	6504	101831
	#Pfams	4800	4461	2554	4261	1084	1260	6485
	#UniProts	25019	27175	7738	15335	2231	4292	26950
≥2 Uniprots, Seqid<90%	#Pfam-Archs	2978	3029	2183	4304	319	659	3263
	#Clusters	16249	24201	3359	9079	407		35305
	#Entries	76839	87536	9995	35545	6470	5856	92098
	#Pfams	3057	3029	1858	3389	319	659	3263
	#UniProts	21556	24858	6850	14361	1531	3926	22931
≥5 Uniprots, Seqid<90%	#Pfam-Archs	847	1011	436	1298	78	275	1280
	#Clusters	1775	2943	560	1893	88		5761
	#Entries	42056	52207	5655	27189	4631	4628	74075
	#Pfams	975	1011	460	1347	78	275	1280
	#UniProts	12364	15381	3511	9937	955	3298	16835
≥10 Uniprots, Seqid<90%	#Pfam-Archs	339	458	166	523	35	166	557
	#Clusters	536	813	196	666	37		1496
	#Entries	28066	36732	3751	19698	3881	3705	56040
	#Pfams	414	458	193	621	35	166	557
	#UniProts	8196	10802	2324	7143	706	2861	12084
≥20 Uniprots, Seqid<90%	#Pfam-Archs	120	178	50	189	12	54	192
	#Clusters	173	270	56	238	12		368
	#Entries	16970	23018	2416	12095	2428	1578	36616
	#Pfams	155	178	74	260	12	54	192
	#UniProts	5010	6862	1425	4598	410	1645	7314

[a] "Same" refers to an interface between two chains or two domains with the same Pfam architecture. #Pfam-Archs is the number of chain-architecture pairs (under "chain") or Pfam-domain pairs (under "domain") in clusters that satisfy the rules in the first column (the minimum number of Uniprots listed and minimum sequence identity < 90%). #Clusters is the number of clusters that satisfy the rules in the first column, and #UniProts is the number of unique UniProt codes in those clusters.

[b] "Diff" refers to an interface between two chains or two domains with different Pfam architectures.

[c] #Pfam-Archs is the number of Pfams which interact with peptides. A peptide is defined as a polypeptide chain with length less than 30 residues. #UniProts is the number of distinct protein-domain UniProts. Seqid is the minimum sequence identity of protein chains in a cluster.

[d] Pfam-DNA/RNA interactions are not clustered. #Pfam-Archs is the number of Pfams that follow the rules in the first column. No sequence identity cutoff is enforced.

[e] Pfam-ligand interactions do not use sequence identity cutoff. Any small molecule except water is considered a ligand. #Pfam-Archs is the number of Pfams which interact with any ligands given the number of Uniprots in the first column.

Supplementary Table 2 | ACT homodimers in different ACT Pfams, proteins and Pfam architectures.

Pfams	UniProt Code	Chain Pfam Arch	#Entries
ACT – ACT	A0A0H2WCF8_BURMA	(ACT)_(Formyl_trans_N)	1
	A0A0H3K044_STAAM	(PDT)_(ACT)	1
	A1A2B5_BIFAA	(PDT)_(ACT)	1
	A1R118_PAEAT	(PDT)_(ACT)	1
	AK1_ARATH	(AA_kinase)_(ACT)_(ACT_7)	1
	AK3_ECOLI	(AA_kinase)_(ACT)_(ACT_7)	2
	ILVH_ECOLI	(ACT)_(ALS_ss_C)	1
	PH4H_HUMAN	(ACT)	1
	Q2YK82_BRUA2	(2-Hacid_dh_C)_(ACT)	1
	Q3SKI5_THIDA	(NAD_binding_3)_(Homoserine_dh)_(ACT)	1
	Q5SIP8_THET8	(ACT)_(Formyl_trans_N)	1
	Q5SK23_THET8	(CBS)_(CBS)_(ACT)	1
	Q6N2L8_RHOPA	(ACT)_(Formyl_trans_N)	1
	Q7MT13_PORGI	(ACT)_(ACT_7)	1
	Q81P63_BACAN	(ACT)	1
	Q81P63_BACAN	(PDH)_(ACT)	2
	Q82UZ2_NITEU	(ACT)_(ALS_ss_C)	1
	Q88LI9_PSEPK	(ACT)_(Formyl_trans_N)	1
	Q8AA93_BACTN;Q8AA93_BACTN	(ACT)_(ACT)	1
	Q8DUV6_STRMU	(PDT)_(ACT)	1
	Q8KBW6_CHLTE	(PDT)_(ACT)	1
	Q97MC0_CLOAB	(AA_kinase)_(ACT)_(ACT_7)	1
	SERA_ECOLI	(2-Hacid_dh_C)_(ACT)	7
	SERA_MYCTU	(2-Hacid_dh_C)_(SDH_beta)_(ACT)	3
	TY3H_RAT	(ACT)	1
	TYRR_ECOLI	(ACT)_(PAS)	1
	Y1403_ARCFU	(ACT)_(Transketolase_C)	1
	**26**	**14**	**37**
ACT – ACT_6	J3QW32_GALSU;J3QW32_GALSU	(ACT_6)_(ACT)	1
	Q9KQ45_VIBCH;Q9KQ45_VIBCH	(ACT_6)_(ACT)	1
	SERB_MYCA1;SERB_MYCA1	(ACT_6)_(ACT)_(HAD)	9
	**3**	**2**	**11**
ACT – ACT_7	AK_CORGL;AK_CORGL	(AA_kinase)_(ACT)_(ACT_7);(ACT)_(ACT_7)	2
	AK_CORGL;AK_CORGL	(ACT)_(ACT_7)	1
	AK_CORGL;Q93C54_CORCT	(AA_kinase)_(ACT)_(ACT_7);(ACT)_(ACT_7)	1
	AK_MYCTU;AK_MYCTU	(ACT)_(ACT_7)	3
	AK_THETH;AK_THETH	(ACT)_(ACT_7)	2
	P74569_SYNY3;P74569_SYNY3	(AA_kinase)_(ACT)_(ACT_7)_(ACT)_(ACT_7)	1
	Q9JYN6_NEIMB;Q9JYN6_NEIMB	(ACT)_(ACT_7)	1
	**6**	**3**	**11**
ACT_3 – ACT_3	Q9KTT6_VIBCH	(ACT_3)_(ACT_7)	1
	**1**	**1**	**1**
ACT_4 – ACT_4	Q8KC80_CHLTE	(ACT_4)	2
	**1**	**1**	**2**

**ACT_5 – ACT_5**	ILVN_ECOLI	(ACT_5)	1
	Q9WZ19_THEMA	(ACT_5)_(ALS_ss_C)	1
	**2**	**2**	**2**
**ACT_6 – ACT_6**	Q87X74_PSESM	(ACT_6)_(Formyl_trans_N)	1
	Q88R07_PSEPK	(ACT_6)_(Formyl_trans_N)	1
	**2**	**1**	**2**
**ACT_7 – ACT_7**	A9CJY8_AGRFC	(ACT_7)	1
	AK1_ARATH	(AA_kinase)_(ACT)_(ACT_7)	1
	AK3_ECOLI	(AA_kinase)_(ACT)_(ACT_7)	2
	AK_METJA	(AA_kinase)_(ACT_7)_(ACT_7)	4
	CAST1_HUMAN;CAST1_HUMAN	(ACT_7)_(ACT_7)	2
	Q7MT13_PORGI	(ACT)_(ACT_7)	1
	Q97MC0_CLOAB	(AA_kinase)_(ACT)_(ACT_7)	1
	Q9KTT6_VIBCH	(ACT_3)_(ACT_7)	1
	**8**	**6**	**13**

Supplementary Table 3 | ErbB proteins in the asymmetric dimer cluster.

ASU	In Cluster			Not In Cluster		
	active	Inactive[a]	Active/inactive	Active[b]	inactive	Active/inactive
Monomer	82	3	-	1	20	-
Dimer or larger	9	1	9	0	32	0
Total	91	4	9	1	51	0

[a] There are 4 asymmetric dimers in the cluster that do not have active monomers. After checking the corresponding literature, 3GOP was dimerized via Juxtamembrane region which activates EGFR kinase, even though no chains are in the active conformation. 3W2R[2] and 5GNK[3] contain inhibitors (W2R and 80U respectively), which form a covalent bond with Cys797, and occupy the ATP binding cleft, with EGFR is a "DFG-in-C-helix-out" inactive conformation. 2R4B (ErbB4) contains the inhibitor GW7, which forms a covalent bond with Cys803[4]. This is similar to EGFR structure 5GNK which has a covalent bond with conserved Cys797.

[b] Only one structure in active conformation is missing the asymmetric dimer. 5JEB contains a novel inhibitor (6JS) of active ErbB2-ErbB3 complex. So the structure is in an active conformation, but does not form the asymmetric dimer in the crystal since the inhibitor overcomes resistance such as growth factors or mutations that stabilize the active form of ErbB2[5].

Detail of the list of entries is provided in "ErbB_AsymDimers_cluster" worksheet of ProtCID_supplementary_data1.xlsx.

Supplementary Table 4 | Ras proteins in α4-α5 dimer cluster.

	All		HRAS (Human+Rat)		KRAS		NRAS		RB11B		RND3	
	Cluster	Not In Cluster	Cluster	Not In Cluster	Cluster	Not In Cluster	Cluster	Not In Cluster	Cluster	Not In Cluster	Cluster	Not In Cluster
GTP	7	7	4	7	2	0					1	0
GNP	71	25	69	18	0	7	1	0	1	0		
GTP+GNP	78	32	73	25	2	7	1	0	1	0	1	0
GDP	17	71	9	14	8	54	0	1	0	2		
Other triphosphates[a]	12	12	9	3	3	8	0	1				
None[b]	1	12	1	12								
**Total**	**108**	**127**	**92**	**54**	**13**	**69**	**1**	**2**	**1**	**2**	**1**	**0**

[a]: Other triphosphates include GCP, DBG, AGN, CAG and GSP.

[b]: None: no phosphate ligands.

Note: GNP is a triphosphate analog. GTP/GNP-bound Ras adopts two interconverting conformations, “inactive” state 1 and “active” state 2, which exhibits dynamic equilibrium[6]. 17 GDP-bound structures also form this α4-α5 dimer. Muratcioglu *et al.* used a suite of tools such as dynamic light scattering (DLS), FRET, and NMR to study multimer formation of KRas in solution[7]. With DLS, the authors observed that the catalytic domain of KRas forms stable dimers, and the GDP-bound KRas dimerizes although to a much lesser extent.

The detail about the list of entries of the cluster is provided in “Ras_Alpha4-5dimers_cluster” worksheet of ProtCID_supplementary_data1.xlsx.

Supplementary Table 5 | 42 Professional Peptide binding domain (PPBD) Pfams containing at least 3 peptide binding human proteins (distinct UniProt codes).

Pfam	Clan	#Entries cluster	#UNP cluster	%Entries Cluster	%UNP Peptides	#HumanUNP	#HumanUNP cluster	#Peptides cluster	#Entries	Surface Area
14-3-3	-	100	15	66	58	8	7	72	152	541
BET	-	9	3	69	43	4	3	9	13	735
BRO1	-	11	3	31	43	6	3	10	35	596
Bromodomain	-	58	19	4	31	40	13	44	1341	434
Cbl_N3	-	26	4	74	80	4	3	18	35	498
CTD_bind	-	12	5	41	56	9	2	6	29	392
Dynein_light	-	19	5	53	45	3	2	19	36	650
HRM	-	11	3	24	15	27	1	11	45	555
Spin-Ssty	-	7	3	64	75	5	3	6	11	370
SWIB	-	40	4	33	50	5	2	26	121	562
WW	-	48	16	26	40	51	10	40	184	416
zf-CW	-	9	6	35	86	7	3	6	26	479
zf-UBR	-	12	3	67	75	6	2	11	18	343
HSP70	Actin_ATPase	32	3	16	12	14	1	24	203	597
Bcl-2	BCLiA	90	19	38	54	12	8	73	240	887
BIR	BIR-like	39	6	32	55	8	5	29	122	368
BRCT	BRCT-like	19	3	28	14	12	2	15	67	309
PTCB-BRCT	BRCT-like	4	3	17	15	7	1	4	24	412
RTT107_BRCT_5	BRCT-like	5	4	50	100	3	2	4	10	267
YEATS	C2	12	6	67	86	4	3	10	18	476
Adap_comp_sub	Cargo_bd_muHD	18	4	43	44	10	1	14	42	537
EF-hand_1	EF_hand	16	6	5	6	74	3	12	350	313
EF-hand_7	EF_hand	91	23	11	11	146	5	78	792	686
EF-hand_8	EF_hand	13	6	7	11	51	2	10	195	361
HORMA	Mad2	12	4	41	57	4	2	7	29	984
PDZ	PDZ-like	142	48	29	30	146	19	117	484	471
PDZ_2	PDZ-like	25	10	24	26	10	1	21	106	322
FERM_C	PH	10	5	24	42	27	1	12	41	498
IRS	PH	9	6	45	46	13	3	8	20	783
PID	PH	15	9	33	45	28	3	11	46	669
WH1	PH	10	5	32	45	11	1	10	31	401
SH2	SH2-like	123	34	29	37	108	21	94	431	442
CAP_GLY	SH3	4	3	11	18	10	2	4	37	461
SH3_1	SH3	87	34	19	27	116	16	65	453	494
SH3_9	SH3	21	12	14	17	100	7	17	145	475
FHA	SMAD-FHA	28	14	31	37	35	6	22	91	407
MATH	TRAF	38	7	72	70	3	5	35	53	465
Chromo	Tudor	47	19	57	66	26	10	27	83	524
MBT	Tudor	8	4	17	36	11	2	8	46	266
PWWP	Tudor	9	4	21	17	25	3	7	42	634
Atg8	Ubiquitin	22	11	30	52	9	4	19	73	595
PHD	zf-FYVE-PHD	91	41	24	53	71	30	36	374	519

Supplementary Table 6 | The 20 Pfams with the largest numbers of interacting ligands.

Pfam ID	#Ligands	#Entries[a]	#Chains	#UniProts	#Entries (Pfam)[b]	#Chains (Pfam)	#UniProts (Pfam)
Pkinase	2090	2884	3851	239	3138	4381	521
Pkinase_Tyr	1057	1418	2273	126	1528	2496	201
Trypsin	954	1890	2289	112	2074	2619	225
Hormone_recep	895	1087	1809	61	1130	1975	138
Asp	618	693	1036	37	1131	1529	68
Bromodomain	539	1248	1854	45	1341	2157	242
Carb_anhydrase	520	826	1052	33	837	1072	72
p450	411	827	1558	127	831	1563	178
V-set	381	1831	4251	175	3914	12351	3677
HATPase_c	374	554	865	68	610	979	124
RVP	345	686	1423	81	753	1559	109
adh_short_C2	342	693	2330	227	807	2812	347
NO_synthase	290	556	1052	10	556	1052	10
Abhydrolase_1	257	398	707	95	476	947	130
PDEase_I	243	291	671	20	292	673	37
PARP	242	264	656	15	268	678	36
Peptidase_C1	240	297	518	52	337	603	98
COesterase	220	325	549	30	352	596	58
Phosphorylase	209	244	314	10	246	316	22
Peptidase_M10	207	290	484	25	296	490	28

[a]:
#entries: the number of PDB entries with bound ligands
#chains: the number of monomers with bound ligands
#uniprots: the number of UniProts with bound ligands
[b]:
#entries(Pfam): the number of PDB entries in the Pfam in the PDB
#chains(Pfam): the number of monomers in the Pfam in the PDB
#uniprots(Pfam): the number of UniProts in the Pfam in the PDB

The full list of Pfams is provided in “PfamLigands” worksheet of ProtCID_supplementary_data2.xlsx.

Supplementary Table 7 | Pfams containing at least four human proteins binding DNAs/RNAs (distinct UniProt codes).

Pfam ID	Clan ID	#PDB	#PDB DnaRna	#UNP PDB	#UNP DnaRna	#HumanUNP	#HumanUNP PDB	#HumanUNP DnaRna
APOBEC_N	CDA	47	10	11	6	10	9	5
bZIP_1	bZIP	49	14	20	10	28	8	5
CBFD_NFYB_HMF	Histone	29	5	29	9	14	9	4
DEAD	P-loop_NTPase	247	72	94	29	71	29	7
DNA_pol_lambd_f	HHH	475	427	13	8	4	7	4
Ets	HTH	60	39	20	16	28	14	11
Forkhead	HTH	31	17	20	11	49	16	10
GTP_EFTU	P-loop_NTPase	358	111	99	33	22	9	4
Helicase_C	P-loop_NTPase	388	111	151	45	109	23	10
Histone	Histone	537	170	96	46	42	32	19
HLH	-	39	22	35	22	115	18	11
HMG_box	HMG-box	63	32	41	13	53	13	5
Homeobox	HTH	175	68	110	43	228	52	20
IMS	-	326	307	15	9	4	4	4
IMS_C	-	327	308	15	9	4	4	4
IMS_HHH	HHH	334	311	18	10	3	5	5
KH_1	KH	89	36	51	21	38	25	11
KOW	KOW	660	609	111	77	9	9	7
LSM	Sm-like	66	27	63	38	20	16	14
MBD	MBD-like	24	19	10	7	9	7	5
Myb_DNA-binding	HTH	140	35	65	16	36	27	4
PAZ	PAZ	57	47	18	9	9	7	5
RHD_dimer	E-set	59	26	17	9	10	7	5
RHD_DNA_bind	P53-like	34	28	13	11	10	6	6
Ribosomal_L7Ae	PELOTA	316	269	74	52	11	7	4
RRM_1	RRM	570	207	240	64	228	146	35
T-box	P53-like	11	8	8	7	17	4	4
WD40	Beta_propeller	597	153	187	60	230	50	10
zf-C2H2	C2H2-zf	175	75	81	13	590	56	6
zf-C4	Zn_Beta_Ribbon	100	75	35	25	46	21	15
zf-CXXC	-	32	20	18	13	12	14	10
zf-H2C2_2	C2H2-zf	298	91	71	18	708	52	10

#PDB: the number of PDB entries
#PDB DnaRna: the number of PDB entries with bound DNA/RNA
#UNP PDB: the number of Uniprots in the PDB
#UNP DnaRna: the number of Uniprots with bound DNA/RNA
#HumanUNP: the number of Uniprots in the Pfam in human proteome
#HumanUNP PDB: the number of human Uniprots in the PDB
#HumanUNP DnaRna: the number of human Uniprots with bound DNA/RNA

The full list of Pfams is provided in “PfamDnaRnas” worksheet of ProtCID_supplementary_data2.xlsx.

Supplementary Table 8 | Numbers of Pfams and UniProts in clans containing more than 30 Pfams

Clan ID	#Pfams						#Uniprots				#Human Uniprots				
	All	PDB	Cluster	peptide	DNA/RNA	ligand	PDB	peptide	DNA/RNA	Ligand	All	PDB	peptide	DNA/RNA	ligand
HTH	256	199	99	21	110	138	1423	26	324	513	570	212	8	69	57
P-loop_NTPase	217	178	118	29	51	165	1428	45	159	1126	872	276	9	18	240
NADP_Rossmann	198	165	121	15	17	162	2189	26	32	1785	343	157	8	4	148
TPR	133	109	78	46	26	87	528	85	36	238	418	134	32	9	56
PDDEXK	127	74	17	2	45	64	168	3	63	115	25	10	1	6	6
E-set	91	68	48	9	1	61	461	17	9	255	438	141	12	5	77
MBB	87	40	21	4	0	39	160	9	0	118	6	1	0	0	1
Zn_Beta_Ribbon	81	48	24	4	20	47	344	4	168	199	213	83	0	28	45
Peptidase_CA	71	48	21	10	2	42	252	24	2	184	173	56	11	0	43
Peptidase_MA	71	42	21	13	0	40	191	39	0	170	110	39	16	0	36
Beta_propeller	71	51	37	14	9	47	360	52	60	211	428	83	21	9	48
OB	70	58	43	5	31	47	471	9	186	147	93	47	1	14	20
AB_hydrolase	70	42	25	4	0	40	465	13	0	368	109	40	3	0	36
Cupin	61	52	40	8	5	51	370	24	10	292	122	50	13	2	45
TIM_barrel	59	55	49	3	4	53	697	6	5	587	41	23	0	0	19
Ubiquitin	59	52	30	9	1	37	375	27	1	127	251	126	14	0	51
PH	56	43	19	11	0	29	233	33	0	94	405	128	14	0	46
Glyco_hydro_tim	54	49	31	6	0	48	586	14	0	498	50	25	4	0	24
Thioredoxin	53	43	25	8	5	32	664	19	10	399	130	83	7	4	50
RNase_H	52	27	18	7	18	26	167	12	57	123	71	17	2	7	12
GT-A	50	29	14	4	0	29	151	4	0	123	116	15	3	0	13
GBD	49	47	23	8	0	40	241	13	0	189	112	32	7	0	26
GT-B	45	33	19	5	4	32	147	5	2	109	51	12	2	0	9
Concanavalin	43	38	26	6	1	36	378	14	1	308	218	41	5	0	30
GPCR_A	41	10	3	3	0	6	97	12	0	76	853	54	9	0	35
Acetyltrans	39	29	15	11	1	27	255	20	1	207	49	22	5	0	20
NTF2	38	32	17	1	1	30	189	5	2	140	16	10	3	1	4
RING	38	26	15	2	2	26	130	2	2	79	339	81	1	1	47
Calycin	37	33	11	3	0	29	160	4	0	125	38	28	2	0	26
C2H2-zf	37	31	6	2	12	28	145	2	29	67	788	81	2	14	32
Met_repress	35	24	7	0	6	11	62	0	13	24	0	1	0	0	0
PKinase	34	23	10	5	4	21	510	49	4	455	529	274	31	1	258
Omega_toxin	34	31	1	0	0	7	124	0	0	10	4	6	0	0	0
Actin_ATPase	32	27	22	5	0	27	213	11	0	177	70	20	1	0	21
HUP	31	29	22	0	11	29	301	0	27	256	40	17	0	1	14

#Pfams: the number of Pfams in PDB. “All” means the number of Pfams in the clan whether they are in the PDB or not; %. “PDB” is the number of Pfams in the clan in the PDB; “cluster” refers to any domain clusters containing at least 2 crystal forms and minimum sequence identity < 90; “peptide” refers to the number of Pfams with bound peptides; “DNA/RNA” refers to the number of Pfams with bound nucleic acid; “ligand” refers to the number of Pfams with bound ligands. The “PDB”, “peptide”, “DNA/RNA” and “ligand” header names are same for “#Uniprots” and “#Human Uniprots”.

#Uniprots: the number of Uniprots.

#Human Uniprots: the number of human Uniprots. “All” means the number of Uniprots in the clan in human proteome.

The full list of clans is provided in “ClanNumbersPfamsUniProts” worksheet of ProtCID_supplementary_data2.xlsx.

Supplementary References

1. Szklarczyk, D. et al. The STRING database in 2017: quality-controlled protein-protein association networks, made broadly accessible. *Nucleic Acids Res.* 45, D362-D368 (2017).
2. Sogabe, S. et al. Structure-Based Approach for the Discovery of Pyrrolo[3,2-d]pyrimidine-Based EGFR T790M/L858R Mutant Inhibitors. *ACS Med. Chem. Lett.* 4, 201-205 (2013).
3. Wang, A. et al. Discovery of (R)-1-(3-(4-Amino-3-(3-chloro-4-(pyridin-2-ylmethoxy)phenyl)-1H-pyrazolo[3,4-d]py rimidin-1-yl)piperidin-1-yl)prop-2-en-1-one (CHMFL-EGFR-202) as a Novel Irreversible EGFR Mutant Kinase Inhibitor with a Distinct Binding Mode. *J. Med. Chem.* 60, 2944-2962 (2017).
4. Wood, E.R. et al. 6-Ethynylthieno[3,2-d]- and 6-ethynylthieno[2,3-d]pyrimidin-4-anilines as tunable covalent modifiers of ErbB kinases. *Proc Natl Acad Sci U S A* 105, 2773-2778 (2008).
5. Novotny, C.J. et al. Overcoming resistance to HER2 inhibitors through state-specific kinase binding. *Nat. Chem. Biol.* 12, 923-930 (2016).
6. Shima, F. et al. Structural basis for conformational dynamics of GTP-bound Ras protein. *J. Biol. Chem.* 285, 22696-22705 (2010).
7. Muratcioglu, S. et al. GTP-Dependent K-Ras Dimerization. *Structure* 23, 1325-1335 (2015).
