# Supplementary material for: ProtCID: a data resource for structural information on protein interactions
Source: Nat Commun. 2020 Feb 5;11:711. doi: 10.1038/s41467-020-14301-4 (PMC7002494; doi:10.1038/s41467-020-14301-4)
Supplement: Supplementary file 3 — Description of Additional Supplementary Files [file 41467_2020_14301_MOESM3_ESM.pdf]

Description of Additional Supplementary Files

File Name: Supplementary Data 1

Description: Detailed data for the homodimer clusters shown in Figure 1, consisting of ErbB proteins, Tyrosine kinases, Ras proteins, and BET proteins. It contains four worksheets, one for each cluster. The ErbB_AsymDimers_cluster worksheet and Ras_Alpha-5dimers_cluster worksheet also contains structures of same proteins but not in the cluster. The column headers are (in order): UniProt, PDB entry; Space group; Crystal form (CF); Chains and symmetry operators for the interface (listed as Chain1(Symm1):Chain2(Symm2)); Surface area in Å2 ; the stoichiometry of the asymmetric unit (ASU); the stoichiometry of the PDB biological assembly (PDBBA); the stoichiometry of the PISA biological assembly (PISABA); whether the dimer in the cluster is present in the asymmetric unit (InASU, where 1=yes, 0=no); whether the dimer in the cluster is present in the PDB biological assembly (InPDB); whether the dimer in the cluster is present in the PISA biological assembly (InPISA); the resolution for X-ray and electron microscopy structures (in Å); a list of ligands (using the PDB's identifiers); mutations in the PDB sequence relative to the UniProt sequence for each chain.

File Name: Supplementary Data 2

Description: Summary data tables for the interactions of Pfam domains with nucleic acids and ligands and the interactions of Pfam clans with nucleic acids, ligands, and peptides. The PfamNucleicAcids page contains these columns (in order): PfamID; ClanID; the number of entries in the PDB that contain the Pfam domain in total (Entries in PDB); the number of entries in the PDB that contain the Pfam domain bound to DNA (Entries with bound DNA); the number of entries in the PDB that contain the Pfam domain bound to RNA (Entries with bound RNA); the number of unique UniProts of the Pfam domain in the PDB in total (UniProts in PDB); the number of unique UniProts of the Pfam in the PDB bound to DNA (UniProts with bound DNA); the number of unique UniProts of the Pfam in the PDB bound to RNA (UniProts with bound RNA); the number of human UniProts that contain the Pfam in the human proteome whether in the PDB or not (UniProts with Pfam in human proteome); the number of unique human UniProts of the Pfam in the PDB in total (Human UniProts with Pfam in PDB); the number of unique human UniProts of the Pfam in the PDB bound to DNA (Human UniProts with bound DNA); the number of unique human UniProts of the Pfam in the PDB bound to RNA (Human UniProts with bound RNA). The PfamLigands page contains these columns (in order): Pfam ID, the number of entries with the Pfam domain in the PDB in total (PDB entries with Pfam); the number of entries with the Pfam domain with bound ligands (PDB Entries with ligands bound to Pfam); the number of chains with the Pfam domain in the PDB in total (PDB chains with Pfam); the number of chains in those entries with the Pfam domain with bound ligands (PDB chains with ligands bound to Pfam); number of unique UniProts with the Pfam in the PDB in total (UniProts in Pfam); the number of unique UniProts that contain the domain with bound ligands (UniProts in Pfam in PDB with ligands); the number of different ligands bound to the Pfam domain in the PDB (Number of distinct ligands). The ClanNucleicAcids page contains these columns (in order): ClanID, the number of Pfams in the clan according to the Pfam website (Pfams in Clan); the number of Pfams in the clan in the PDB (Pfams in Clan in PDB); the number of Pfams in the clan with bound DNA and/or RNA (Pfams with bound nucleic acid in PDB); the number of unique UniProts in the clan in the PDB in total (UniProts with Clan domain in PDB); the number of unique UniProts in the clan with bound DNA and/or RNA (UniProts with bound nucleic acid in PDB); the number of Pfams in the clan in human proteome (Pfams in Clan in Human proteome); the number of Pfams in the clan with human UniProts in the PDB (Pfams with human UniProts in PDB); the number of Pfams in the clan with human UniProts in the PDB with bound nucleic acid (Pfams with human UniProts with bound nucleic acid in PDB); the number of unique UniProts in the clan in human proteome (UniProts in Clan in human proteome); the number of unique UniProts in the clan in the PDB (Human UniProts in Clan in

PDB); the number of human UniProts in the clan in the PDB with bound nucleic acid (Human UniProts with bound nucleic acid in PDB). The ClanNumbersPfamsUniProts page contains these columns (in order): ClanID; the number of Pfams in the Clan according to the Pfam website (Pfam in Clan); the number of Pfams in the Clan that are in the PDB (Pfams in Clan in PDB); the number of Pfams in the Clan that have clusters with at least two distinct crystal forms and minimum sequence identity less than 90% in ProtCID (Pfams with ProtCID domain clusters); the number of Pfams in the Clan with bound peptides (Pfams with bound peptides in PDB); the number of Pfams in the Clan with bound nucleic acid (Pfams with bound nucleic acid in PDB); the number of Pfams in the Clan with bound ligands (Pfams with bound ligands in PDB); the number of UniProts in the PDB structures of the Clan (UniProts in PDB); the number of UniProts in the PDB structures of the Clan that bind peptides (UniProts with bound peptides in PDB); the number of UniProts in the Clan in the PDB with bound nucleic acids (UniProts with bound nucleic acid in PDB); the number of UniProts in the Clan in the PDB with bound ligands (UniProts with bound ligands); the number of human UniProts that contain a Pfam in the Clan (UniProts in Clan in human proteome); the number of human UniProts in the Clan that are in the PDB (Human UniProts in Clan in PDB); the number of human UniProts in the Clan with bound peptides that are in the PDB (Human UniProts with bound peptides in PDB); the number of human UniProts in the Clan with bound nucleic acids that are in the PDB (Human UniProts with bound nucleic acid in PDB); the number of human UniProts in the Clan with bound ligands that are in the PDB (Human UniProts with bound ligands in PDB).
